# *Escherichia coli* Contamination across Multiple Environmental Compartments (Soil, Hands, Drinking Water, and Handwashing Water) in Urban Harare: Correlations and Risk Factors

**DOI:** 10.4269/ajtmh.17-0521

**Published:** 2018-01-22

**Authors:** Tala Navab-Daneshmand, Max N. D. Friedrich, Marja Gächter, Maria Camila Montealegre, Linn S. Mlambo, Tamuka Nhiwatiwa, Hans-Joachim Mosler, Timothy R. Julian

**Affiliations:** 1 School of Chemical, Biological, and Environmental Engineering, Oregon State University, Corvallis, Oregon;; 2 Department of Environmental Microbiology, Eawag, Swiss Federal Institute of Aquatic Science and Technology, Dübendorf, Switzerland;; 3 Department of Environmental Social Sciences, Eawag, Swiss Federal Institute of Aquatic Science and Technology, Dübendorf, Switzerland;; 4 Department of Biological Sciences, University of Zimbabwe, Harare, Zimbabwe

## Abstract

*Escherichia coli* pathotypes (i.e., enteropathogenic and enterotoxigenic) have been identified among the pathogens most responsible for moderate-to-severe diarrhea in low- and middle-income countries (LMICs). Pathogenic *E. coli* are transmitted from infected human or animal feces to new susceptible hosts via environmental reservoirs such as hands, water, and soil. Commensal *E. coli*, which includes nonpathogenic *E. coli* strains, are widely used as fecal bacteria indicator, with their presence associated with increased likelihood of enteric pathogens and/or diarrheal disease. In this study, we investigated *E. coli* contamination in environmental reservoirs within households (*N* = 142) in high-population density communities of Harare, Zimbabwe. We further assessed the interconnectedness of the environmental compartments by investigating associations between, and household-level risk factors for, *E. coli* contamination. From the data we collected, the source and risk factors for *E. coli* contamination are not readily apparent. One notable exception is the presence of running tap water on the household plot, which is associated with significantly less *E. coli* contamination of drinking water, handwashing water, and hands after handwashing. In addition, *E. coli* levels on hands after washing are significantly associated with handwashing water contamination, hand contamination before washing, and diarrhea incidence. Finally, we observed that animal ownership increases *E. coli* contamination in soil, and *E. coli* in soil are correlated with contamination on hands before washing. This study highlights the complexity of *E. coli* contamination in household environments within LMICs. More, larger, studies are needed to better identify sources and exposure pathways of *E. coli*—and enteric pathogens generally—to identify effective interventions.

## INTRODUCTION

Gastrointestinal diseases cause an estimated 5–700,000 deaths in children aged less than 5 years annually.^1^ Recent studies have identified a subset of enteric pathogens (enterotoxigenic and enteropathogenic *Escherichia coli*, *Shigella* spp., rotavirus, calicivirus, and *Cryptosporidium* spp.) as the leading causes of moderate-to-severe diarrhea and/or mortality in low- and middle-income countries (LMICs).^2,3^ These pathogens are likely transmitted from human or animal feces to susceptible hosts through interactions with food and environmental compartments (i.e., flies, hands, soil, surfaces, and water).^3–5^ Fecal indicator bacteria, including *E. coli*, are widely used as indicators to study the sources and fate of fecal contamination in the environment. The presence of *E. coli* in drinking water, for example, is associated with increased risk of both enteric pathogens and diarrheal disease, generally.^6–8^ Research to date largely focuses on the role of food and water in the transmission of enteric bacteria, but recent evidence has highlighted the potential importance of other compartments including hands and soil.^9–13^

There is increasing awareness of the role of environmental compartments such as hands, surfaces, and soil in the transmission of enteric bacteria. Hands, in particular, are important compartments in diarrheal disease transmission. An estimated 297,000 diarrheal deaths could be avoided with improved hygiene, equivalent to approximately 5.5% of all causes of death for children aged less than 5 years.^14^ It is therefore unsurprising that high levels of microbial indicators of fecal contamination—as well as enteric pathogens—have been reported on the hands of people in LMICs.^12,15,16^ Even after a handwashing event, *E. coli* contamination on hands can increase 2–3 log_10_ colony forming units (CFU) per two hands within only a few minutes when typical household activities (e.g., cleaning dishes, preparing food, sweeping, and bathing) are performed.^17–19^ The cause of the rapid contamination is unclear, but an exposure model by Julian and Pickering^20^ suggested that *E. coli* contamination of dirt and sand may contribute substantially to hand contamination.

Soil and surfaces within households are also important exposure pathways for diarrheal disease in LMICs. Several studies have reported high levels of fecal indicators on surfaces and in the soil of households.^10,13,21–24^ The sources of the fecal contamination are unclear, but evidence suggests both human and animal sources.^10,13,25^ Fecal contamination of soil is likely due to some combination of open defecation, inadequate infant and child fecal management, inadequate disposal of wastewater, and inadequate animal fecal management.^16,26–31^ Moreover, fecal indicator bacteria may be indigenous and/or naturalized in soil in tropical climates.^27,32,33^ Finally, soil is important because of the potential for soil ingestion, which has been reported in multiple studies (i.e., in Bangladesh, Zimbabwe, Kenya, and Taiwan).^34–38^ Understanding the role of hands, surfaces, and soil in the transmission of enteric bacteria is critical to help inform effective interventions.^4^

There is increasing evidence of the interconnectedness of the environmental compartments in pathogen transmission in LMICs. Although both hand and soil contamination can directly impact human health via, for example, hand-to-mouth contacts and soil ingestion, these compartments can also transfer pathogens to other compartments (e.g., food and drinking water). For example, the decline in stored drinking water quality relative to the source drinking water is often attributed to water contacts with contaminated hands or utensils.^39–42^ Further evidence of this is supported by findings that stored drinking water contamination is associated with contamination on hands with *E. coli* virulence genes, fecal indicator bacteria, and enteric viruses.^12,15^ Reducing opportunities for hand contact with water (e.g., by using containers with smaller openings) is associated with reductions in fecal contamination.^41,43^

The present study investigates associations in *E. coli* contamination between environmental compartments (hands, soil, drinking water, and handwashing water) in high-population density communities of Harare, Zimbabwe. The objective is to identify risk factors for *E. coli* contamination in drinking and handwashing water, soil, and hands. We modeled associations between sanitation, hygiene, and diarrhea incidence on *E. coli* levels in soil, water (drinking and handwashing), and on hands (before and after handwashing) using both microbiological sampling for *E. coli*, as well as household-level survey and observation data. Study outcomes highlight the interconnectedness between compartments and identify likely household-level factors that influence environmental *E. coli* contamination.

## MATERIALS AND METHODS

### Conceptual framework.

**Figure 1.**
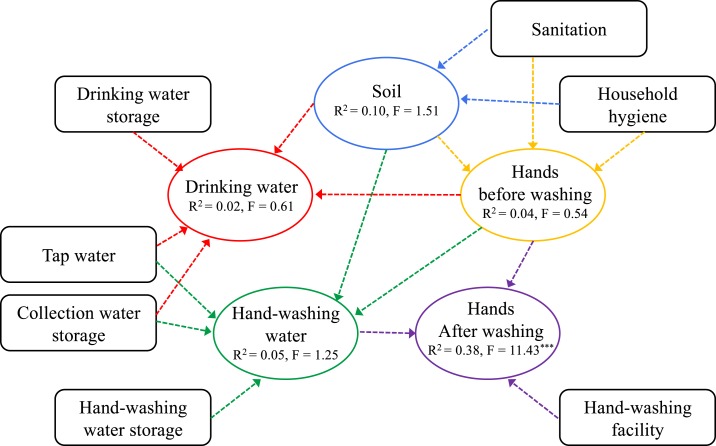
Conceptual framework of the study. Oval boxes represent compartments for enteric bacteria (i.e., hands, soil, and water) and square boxes represent household survey data. Arrows signify the potential correlations of fecal contamination between different compartments and households’ characteristics. The impact of diarrhea incidents and asset index rankings on each compartment—not illustrated here—was also studied. Values for *R*-squared and *F* statistics from the regression models are displayed for each compartment. Each color denotes the potential pathways hypothesized for enteric bacterial transmission to a certain compartment. *** indicates significance at the 1% level. This figure appears in color at www.ajtmh.org.

The conceptual framework of our study is that *E. coli* contamination within environmental compartments (soil, drinking water, handwashing water, hands before handwashing, and hands after handwashing) is influenced by environmental contamination within other compartments and is mediated by sanitation, hygiene, and health (Figure 1). For example, *E. coli* contamination in hands after washing (Figure 1) is influenced by *E. coli* contamination of handwashing water and hands before handwashing, as well as handwashing facility characteristics. Other compartments (i.e., soil) and household-level factors (i.e., sanitation, household hygiene, presence of running tap water, and handwashing water collection and storage conditions) indirectly influence drinking water through impacts on other compartments. The impact of diarrhea incidence and asset index rankings on each compartment—not illustrated in Figure 1—was also studied. The conceptual framework formed the basis for the data collection and analysis.

### Study site and sampling frame.

This study was conducted in high-population density communities of urban Harare (Zimbabwe) in January and February 2016 and embedded within a cluster-randomized controlled trial of 580 households powered to evaluate the effects of a hand washing campaign in collaboration with the Ministry of Primary and Secondary Education, the Ministry of Health and the City Health Authority of Harare.^44^ In this study, 142 households were enrolled, or 25% of the total enrolled in the larger trial, based on the logistical constraints (timing, sample transport, and sample processing) associated with the more intense environmental sampling. Participants were recruited through random route sampling. In brief, from a randomly selected junction in each of the 20 clusters, every fifth household was visited. Households were enrolled if 1) at least one child attended the local primary school and 2) the primary caregiver provided informed written consent. Households that were nonresponsive, ineligible, and unwilling to participate were replaced by the fifth next household on the sampling route. The study was reviewed and approved by the Ethics Commission of the Swiss Federal Institute of Technology Zurich (ETH Zurich, Zurich, Switzerland) and the Research Council of Zimbabwe.

### Household interview data.

Data were collected via personal interviews with the primary caregiver and spot check observations by local enumerators. The interviews included questions on demographics; diarrhea incidence (loose, watery stools) in the past 7 days; assets as a proxy for socioeconomic status; and water, sanitation, and hygiene (WASH) infrastructure and practices (Table 1).

**Table 1:** Characteristics of households

Characteristics	All households (*N* = 142)		With running tap water (*N* = 98)		Without running tap water (*N* = 44)	
	*n*	Percent/mean ± SD	*n*	Percent/mean ± SD	*n*	Percent/mean ± SD
Diarrhea incidence						
Diarrhea incidence in the past 7 days	27	19	20	20	7	15
Asset index ranking						
Low	26	18	19	19	7	15
Medium	85	59	60	61	25	56
High	31	21	19	19	12	27
Sanitation						
Presence of animals in household	17	12	8	8	9	20
Toilet structure completeness additive index	142	0.41 ± 0.68	98	0.36 ± 0.60	44	0.52 ± 0.82
Toilet cleanliness additive index	142	0.78 ± 0.74	98	0.77 ± 0.72	44	0.82 ± 0.81
Toilet located outside the house	76	53	48	49	28	63
Household hygiene						
Presence of trash inside/outside the house	60	42	42	42	18	40
Presence of flies inside the house	90	63	60	61	30	68
Handwashing facility						
Handwashing facility (after contact with feces) outside the house	108	76	76	77	32	72
Absence of soap and water	53	37	27	27	26	59
Tap water						
Absence of running tap water	44	31	–	–	–	–
Collection water storage						
Presence of visible dirt inside	–	–	–	–	10	22
Container not covered or closed, with large opening or spigot	–	–	–	–	37	84
Handwashing water storage						
Presence of visible dirt inside	–	–	–	–	12	27
Container not covered or closed, with large opening or spigot	–	–	–	–	37	84
Drinking water storage						
Presence of visible dirt inside	–	–	–	–	7	15
Container not covered or closed, with large opening or spigot	–	–	–	–	36	81

SD = standard deviation.

### Soil samples.

#### Collection.

Soil samples (*N* = 142) were obtained from each household from an outdoor location closest to the entrance of the house where there was no visible feces, food, or trash. If there was more than one entrance to the house, the entrance closest to the food preparation area was chosen. All soil samples were collected from the surface (< 2 cm deep) of an area approximately 20 × 20 cm. A sterile plastic spoon was used to collect more than 80 g of soil in a 120-mL Whirl-Pack^®^ (Sigma-Aldrich, Buchs, Switzerland) sampling bag. Soil samples were stored on ice and transported to the laboratory at the University of Zimbabwe where they were processed within 6 hours.

#### Total solids.

Total solids of the soil samples were measured following a method adapted from Colorado University Extension using a microwave oven.^13,45^ In brief, soil weights were recorded for 5 g soil samples, and then the soil was subjected to repeated 10-second intervals of microwaving. Final dry weight was determined when three consecutive measurements differed by < 0.1 g. Moisture content calculations were based on the difference between initial and final soil sample weights.

#### *E. coli* enumeration.

To elute *E. coli* from soil samples, 5 ± 0.25 g of soil was added to 30 mL phosphate buffered saline (PBS) in a 120 mL Whirl-Pack bag. PBS was made in the laboratory at the University of Zimbabwe using 8 g NaCl, 0.2 g KCl, 1.44 g Na_2_HPO_4_, and 0.24 g KH_2_PO_4_ in 1 L of dechlorinated bottled water (18 mg sodium thiosulfate per L of Shoppers Choice purified bottled water; OK Zimbabwe Limited, Harare, Zimbabwe). The mixture was shaken by hand for 2 minutes and then allowed to settle for 15 ± 3 minutes. For *E. coli* quantification, 1 mL of the soil supernatant from both undiluted and 1/50 dilution in PBS was plated onto compact dry plates (Compact Dry^™^ EC, VWR, Vienna, Austria). The plates were incubated at 37°C for 24 hours following the manufacturer’s instructions. Duplicate samples were tested for 10% of the households. One negative field control was processed per sampling day. Results were calculated as CFU per gram of total solids (CFU/g-TS). The lower and upper detection limits were 0.89 log_10_ CFU/g-TS and 4.76 log_10_ CFU/g-TS, respectively.

#### *E. coli* isolation.

Presumptive *E. coli* colonies were isolated from a randomly selected subset of soil samples (*N* = 49). In brief, 1 mL of the soil supernatant from both 1× and 1/50 dilutions in PBS was filtered on a Whatman^®^ cellulose acetate membrane filter (0.45 μm thickness, 47 mm diameter; Sigma-Aldrich). The filters were placed on tryptone bile glucuronic (TBX) media (Thermo Fisher Scientific, Waltham, MA) and incubated at 37°C for 18–24 hours per the manufacturer’s instructions. Blue-green presumptive *E. coli* colonies (three per sample) were isolated and grown overnight at 37°C in 1 mL LB broth. From the overnight culture, 100 μL was added to 1 mL glycerol-tryptone soy broth (TSB) solution (30% glycerol–70% TSB made with 30 g TSB media in 1 L dechlorinated bottled water) and kept at −20°C for up to 40 days before shipping to Eawag (Dübendorf, Switzerland) for further processing. At Eawag, presumptive *E. coli* isolates (*N* = 30) were identified with API-20E kits (bioMérieux, Genève, Switzerland) following the manufacturer’s instructions.

### Water samples.

#### Collection.

Water samples (*N* = 244) were obtained from 142 households. In 102 households (72%), there were separate sources of water for drinking and handwashing; in these households, water samples were collected from both sources. The other 40 households (28%) used a single water source for both drinking and handwashing. Samples were obtained in 125-mL polypropylene sample bottles (Semadeni, Ostermundigen, Switzerland) containing sodium thiosulfate for dechlorination with a final concentration of 18 mg/L. Every sampling day, one bottle was filled with Shoppers Choice bottled water also treated with 18 mg/L sodium thiosulfate in the laboratory, transported during sampling on the field alongside other samples, and returned to the laboratory for microbial analyses as a field control. Water containers were stored on ice for up to 6 hours before processing in the laboratory at the University of Zimbabwe.

#### *E. coli* enumeration.

For *E. coli* analysis, both 1 and 100 mL samples were analyzed. The 1 mL sample was pipetted directly on a compact dry plate. The 100 mL of water sample was filtered using 0.45 μm cellulose acetate membrane filters (47 mm diameter; Sigma-Aldrich), and the filter was placed on the compact dry plate. The plates were incubated at 37°C for 24 hours as per the manufacturer’s instructions. Duplicate samples were tested for 10% of the households. Negative field controls were processed in parallel for each sampling day. The lower and upper detection limits were 0 log_10_ CFU/100 mL and 4.3 log_10_ CFU/100 mL, respectively.

#### *E. coli* isolation.

From a randomly selected subset of water samples (*N* = 48), presumptive *E. coli* colonies were isolated. One hundred milliliter of the water sample was filtered on a Whatman cellulose acetate membrane filter (0.45 μm thickness, 47 mm diameter; Sigma-Aldrich). The filters were placed on TBX plates, incubated at 37°C for 24 hours, and the colonies were isolated following the methods previously described for soil. At Eawag, presumptive *E. coli* isolates (*N* = 25 for drinking water, *N* = 9 for handwashing water) were identified with API-20E kits (bioMérieux) following the manufacturer’s instructions.

### Hand rinse samples.

Contamination of hands before and after handwashing was measured using hand rinse samples.^46,47^ To collect the “hands before washing” sample, the caregiver’s left or right was selected randomly. The selected hand was placed in a 2,040-mL Whirl-Pack Sampling bag (NASCO Corp., Fort Atkinson, WI) containing 350 mL bottled water treated with 17.5 mg/L sodium thiosulfate. The bag was fastened on the caregiver’s wrist. The hands were massaged as described elsewhere by Friedrich et al.^46^ Bags containing hand rinse samples were placed on ice for up to 6 hours before processing in the laboratory at the University of Zimbabwe. Next, the caregiver was asked to wash their hands in their usual manner before handling food or after contact with feces. To collect the “hands after washing” sample, the other hand of the caregiver that was not previously sampled was used following the method described previously. The lower and upper detection limits were 0.54 log_10_ CFU/hand and 4.94 log_10_ CFU/hand for hands before handwashing and 0.54 log_10_ CFU/hand and 3.94 log_10_ CFU/hand for hands after handwashing, respectively.

### Virulence genes detection.

To assess whether virulent *E. coli* strains were circulating in the community at the time of sampling, a randomly selected subset of 45 *E. coli* isolates (*N* = 3 for handwashing water, *N* = 9 for drinking water, *N* = 10 for soil, *N* = 14 for human feces, and *N* = 9 for chicken feces) were further characterized for the presence of six virulence-associated gene markers: *bfp* (bundle forming pilus), *eae* (intimin), *lt* (heat labile enterotoxin), *st* (heat stable enterotoxin), *aat* (anti-aggregation protein transporter gene), and *aaiC* (secreted protein) using a previously described protocol.^48^ Human and chicken feces were included as presumptive sources of fecal contamination into the environment. A subset of *E. coli* isolates from human and chicken feces were also confirmed using API-20E to verify the selectivity of our *E. coli* isolation method from environmental reservoirs relative to feces. Methods for the collection of human and chicken feces are available in the Supplemental Data.

### Data analyses.

All statistical analyses were performed using R software (R Statistical Software, R Foundation for Statistical Computing, Vienna, Austria).^48^ Statistical significance was defined using an α = 0.05. To investigate relationships between environmental *E. coli* contamination and survey data, linear regression models were used. *E. coli* contamination was modeled as a continuous variable of log_10_ concentration (CFU/g dry soil, CFU/hand, or CFU/100 mL). When *E. coli* concentrations were less than or greater than the limit of detections (as described in Materials and Methods), the concentration was assumed to be equal to the limit of detection. Pearson correlation coefficients were calculated for the duplicate *E. coli* concentrations in handwashing water, drinking water, and soil sample. Survey data were modeled as binary variables where the presence of a risk factor was coded as 1, and the absence of the risk factor was coded as 0. For example, the absence of running tap water is a risk and received a score of 1, so households with running tap water received a score of 0.

Based on the number of assets (electricity, refrigerator, television, radio, mobile phone, van, bicycle, wardrobe, table, chair, and clock) households were assigned an asset index that was categorized into three variables: low, medium, and high. Asset index rankings were determined by subsetting the distribution of the total number of assets (between 0 and 11) such that approximately a quarter of households were classified as low, a quarter as high, and the middle 50% as medium. Based on the data, low was classified as less than 7 items, medium as 7–9, and high as more than 10. Medium asset index ranking was set as the reference category in the regression models.

Survey data for some WASH characteristics were aggregated into indices when possible. For example, sanitation indicators included a toilet structure completeness index, indicated by an additive score of whether the toilet had a complete roof, complete walls, full door, and/or concrete slab (range = 0–4). Similarly, toilet cleanliness was based on an additive score based on the evidence of fresh feces in the toilet bowl, visible feces in the toilet area, water or urine on the toilet, and absence of toilet cleaning items (range = 0–4). The corresponding positive attributes for the two indices were given scores of 0 for each item included in the analyses.

Because households with running tap water were identified as outliers in initial comparisons, running tap water as an indicator was assessed independently. Specifically, independent Student’s *t* tests compared *E. coli* levels in handwashing water, drinking water, soil, hands before washing, hands after washing between households with and without running tap water. Independent Student’s *t* tests were also conducted to assess the impact of having separate sources of water for drinking and handwashing on *E. coli* concentrations in water samples.

**Figure 2.**
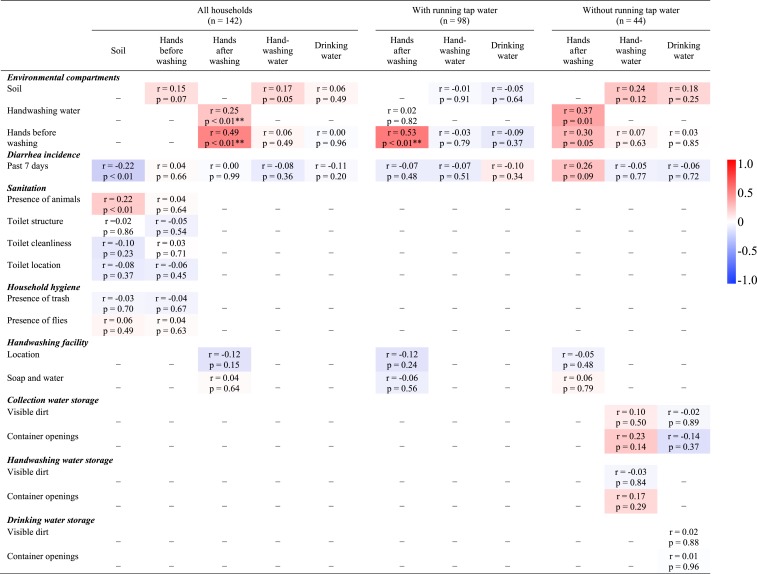
Correlation matrix for *Escherichia coli* levels in soil, hands (before and after washing), and water (drinking and handwashing) for all households as well as households with and without running tap water. Spearman rank (*r*) and *P* values are shown for the correlations included in the analyses. The scale bar shows the correlation coefficient (Spearman rank). Red represents positive correlations and blue represents negative correlations. For interpretation of the references to color in this figure legend, the reader is referred to the web version of this article at www.ajtmh.org.

To investigate associations between environmental compartments, both correlation analyses and multivariate regression analyses were used. Correlation analyses were performed between soil, water (drinking and handwashing), and hand (before and after washing) samples, and the survey data. Again, households with and without running tap water were analyzed separately for handwashing water, drinking water, and hands after washing in households with and without running tap water. The correlations for soil and hands before washing were only performed for all households. Figure 2 shows the correlations included in the analyses for *E. coli* levels in soil, hands (before and after washing), and water (drinking and handwashing) for all households as well as households with and without running tap water. A Holm–Bonferroni correction was applied to control the family-wise error rates for significant correlations.^49^ The adjusted *P* value for Holm–Bonferroni corrections are reported as *adj. P*. The strength of Spearman ρ correlations was interpreted as weak (*r*_s_ < 0.20), moderate (0.20 < *r*_s_ < 0.50), or strong (0.50 < *r*_s_ < 0.80).^50^ Multivariate regression analyses investigated associations between environmental compartments and household-level characteristics (from surveys and observations) based on the conceptual framework (Figure 1, Table 3). Specifically, five independent models were designed to assess associations for soil, hands (before and after washing), and water (drinking and handwashing) for all households. In addition, six independent models were designed to assess associations for hands after washing, handwashing water, and drinking water for both households with and without running tap water.

## RESULTS

### Household characteristics.

Surveys and observations highlighted the sanitation, hygiene, handwashing, and diarrhea incidence characteristics of households (Table 1). The majority (69%) had running tap water. In 19% of the 142 households, there was at least one diarrhea episode (i.e., loose or watery stools) reported in the 7 days before the interview. Household ownership of animals (chicken, cattle, dog, rabbit, pigeon, or turtle) on the household plot was relatively low (12%). All the 142 households had toilets on the household plot. In about half of households, toilets were outside the house (54%), and trash (42%) and flies (63%) were also observed in some households. The handwashing facility for use after contact with feces was located outside the house in 76% of the households. Handwashing facilities of the households often had no soap (27%) or water (13%) present at the time of the interview. More than one-third (37%) of the handwashing facilities had no soap and no water. Of the 44 (31%) households without running tap water, many had visible dirt in their storage containers. Visible dirt was present in 23%, 27%, and 16% of the storage containers use for collecting water, storing handwashing water, and storing drinking water, respectively.

### *E. coli* contamination.

Often, the samples (handwashing water (40%), drinking water (63%), soil (63%), hands before handwashing (75%), and hands after handwashing (73%)) were contaminated with presumptive *E. coli* above the lower limits of detection (Table 2). Average (±standard deviation [SD]) concentrations were 0.61 (±0.92) and 0.77 (±0.86) log_10_ CFU/100 mL in handwashing and drinking water, respectively; 1.52 (±0.94) and 1.32 (±0.80) log_10_ CFU/hand for hands before and after handwashing, respectively; and 1.62 (±0.96) log_10_ CFU/g dry solids in soil. Duplicate samples were generally consistent; the Pearson correlation coefficients amongst duplicates were 1.00 for handwashing water, 0.90 for drinking water, and 0.93 for soil samples. Average (±SD) moisture content of soil was 10.2 (±5.1)% and correlated significantly with *E. coli* concentrations in soil (Spearman’s ρ = 0.36, *P* < 0.001, *adj. P* < 0.01).

**Table 2:** *Escherichia coli* concentration in environmental compartments and on hands for all households and for households with and without running tap water

		All households (*N* = 142)				With running tap water (*N* = 98)	Without running tap water (*N* = 44)		
Variable	Units	Below LLOD (%)*	Mean ± SD	Median	Range	Mean ± SD	Mean ± SD	*t*	*df*
Handwashing water	CFU/100 mL	60	0.61 ± 0.92	0.00	(0.00–3.93)	0.23 ± 0.57	1.48 ± 0.97	7.95**	56.85
Drinking water	CFU/100 mL	37	0.77 ± 0.86	0.48	(0.00–3.93)	0.56 ± 0.76	1.24 ± 0.91	4.29**	70.63
Soil	CFU/g dry solids	37	1.62 ± 0.96	1.10	(0.89–4.76)	1.54 ± 0.91	1.80 ± 1.07	1.40	72.42
Hands before washing	CFU/hand	25	1.52 ± 0.94	1.35	(0.54–4.58)	1.46 ± 0.93	1.64 ± 0.97	1.04	79.60
Hands after washing	CFU/hand	27	1.32 ± 0.80	1.09	(0.54–3.94)	1.21 ± 0.77	1.56 ± 0.81	2.46*	79.65

The *t* values and degrees of freedom are reported for *t* tests on presence of running tap water. All concentration values are in log_10_ format. SD = standard deviation.

* Lower limit of detection; * *P* < 0.05; ** *P* < 0.01.

Presumptive *E. coli* isolates from TBX plates were confirmed using API-20E test kit (bioMérieux). Of the 154 tested, 143 isolates (93%) were identified as *E. coli* and among the *E. coli*, 90% were identified with excellent, very good, or good confidence (confidence level > 90.0%), 8% with acceptable confidence (confidence level 80.0–89.9%), and 2% with low confidence. Specifically, in 83% of the soil (25/30), 78% of handwashing water (7/9), 80% of drinking water (20/25), 96% of human feces (52/54), and 100% of chicken feces (35/35) isolates, *E. coli* was identified with a confidence level above 80%. The proportion of each sample type with confidence level above 80% was not statistically different from any other as shown using χ^2^ tests, adjusted for multiple comparisons using false discovery rate method (*adj. P* > 0.05 for all pairs). The non-*E. coli* isolates (7%) were *Enterobacter cloacae*, *Enterobacter asburiae*, *Kluyvera* spp., *Citrobacter koseri*/*amalonaticus*, or *Leclercia adecarboxylata*. Unlike *E. coli* isolates, these isolates generally fermented sucrose and amygdalin and tested negative for lysine decarboxylase. Overall, TBX media was sufficiently selective for *E. coli* in this environment.

Based on the results of the virulence gene screening, two of the isolates (one from human feces and one from drinking water) were positive for the presence of the *aaiC* gene, demonstrating the presence of enteroaggregative *E. coli* (EAEC) in this environment.

### Sources for drinking and handwashing water.

As mentioned earlier, 72% of the households used separate sources of water for drinking and handwashing and 28% of the households used a single water source for both drinking and handwashing. The source of the water used for handwashing and drinking (same source versus different source) did not significantly impact water quality for handwashing water (*P* = 0.10, based on Student’s *t* tests) or drinking water (*P* = 0.61, based on Student’s *t* tests).

### Presence of running tap water.

The presence of running tap water in households significantly reduced *E. coli* levels in handwashing and drinking water (Table 2). Specifically, the mean *E. coli* concentration in households with running tap water was 1.25 log_10_ CFU/100 mL and 0.68 log_10_ CFU/100 mL lower in handwashing water and drinking water (*P* < 0.001, based on Student’s *t* tests), respectively. In addition, *E. coli* contamination on hands after washing were 0.35 log_10_ CFU/hand lower in households with running tap water (*P* = 0.02, based on Student’s *t* tests). The presence of running tap water, however, did not have a significant impact on *E. coli* levels in soil or on hands before handwashing.

### *E. coli* contamination correlations between environmental compartments.

Correlation analyses showed no-to-moderate associations between *E. coli* contamination in environmental compartments and household-level factors (Figure 2). In households with running tap water, *E. coli* on hands after washing was strongly associated with contamination on hands before washing (Spearman’s ρ = 0.53, *P* < 0.001, *adj. P* < 0.01). In households without running tap water, *E. coli* on hands after washing was moderately associated with contamination on hands before washing (Spearman’s ρ = 0.30, *P* = 0.05, *adj. P* > 0.05), and in handwashing water (Spearman’s ρ = 0.37, *P* = 0.01, *adj. P* > 0.05). Across all households, *E. coli* levels in soil samples were moderately associated with the presence of animals (Spearman’s ρ = 0.22, *P* = 0.008, *adj. P* > 0.05; Figure 2), and inversely correlated with diarrhea incidence (Spearman’s ρ = −0.22, *P* = 0.009, *adj. P* > 0.1).

### *E. coli* contamination regression models.

Among households with running tap water, *E. coli* contamination in environmental compartments is only weakly or slightly explained by the household-level factors and contamination in other compartments we investigated (Table 3; *P* values are presented in Supplemental Table 1). Specifically, *E. coli* contamination in water (both handwashing water and drinking water) is not explainable by the factors investigated (*R*^2^ < 0.10). The most predictive model observed is for *E. coli* on hands after washing (*R*^2^ = 0.47, *P* < 0.001). This is largely due to the strong correlation with *E. coli* on hands before washing (β = 0.59, standard error [SE] = 0.07, *P* < 0.001).

**Table 3:** Regression coefficients of environmental compartments and hand hygiene models, reported as standardized coefficients (standard error)

	All households					With running tap water			Without running tap water		
	Soil	Hands before washing	Hands after washing	Handwashing water	Drinking water	Hands after washing	Handwashing water	Drinking water	Hands after washing	Handwashing water	Drinking water
Observations	140	140	139	140	140	96	97	97	43	42	42
Constant	1.67** (0.21)	1.14** (0.25)	0.61** (0.17)	0.26 (0.20)	0.77** (0.19)	0.34 (0.19)	0.24 (0.20)	0.90** (0.26)	0.38 (0.43)	0.43 (0.59)	1.01* (0.57)
Environmental compartments											
Soil	–	0.17* **(0.09)**	–	0.14 (0.08)	0.03 (0.08)	–	0.00 (0.07)	−0.10 (0.09)	–	0.12 (0.17)	0.23 (0.16)
Handwashing water	–	–	0.13* **(0.06)**	–	–	−0.03 (0.11)	–	–	0.25 (0.13)	–	–
Hands before washing	–	–	0.50** (0.06)	0.08 (0.09)	−0.00 (0.08)	0.59** (0.07)	0.01 (0.07)	−0.08 (0.09)	0.33* **(0.13)**	0.09 (0.18)	0.03 (0.17)
Diarrhea incidence											
Past 7 days	−0.40 (0.22)	0.09 (0.21)	−0.09 (0.15)	−0.12 (0.20)	−0.21 (0.19)	−0.13 (0.16)	−0.13 (0.15)	−0.31 (0.20)	0.17 (0.33)	−0.17 (0.47)	0.23 (0.44)
Asset ranking											
High	0.19 (0.22)	0.05 (0.22)	−0.10 (0.15)	0.17 (0.20)	0.13 (0.19)	0.05 (0.21)	0.06 (0.19)	0.06 (0.25)	0.22 (0.28)	0.11 (0.38)	0.03 (0.35)
Low	0.07 (0.22)	0.08 (0.21)	−0.12 (0.15)	0.01 (0.21)	−0.13 (0.20)	0.31 (0.17)	−0.01 (0.16)	−0.02 (0.21)	0.11 (0.34)	0.06 (0.51)	−0.62 (0.44)
Sanitation											
Presence of animals	0.48 (0.26)	0.06 (0.26)	–	–	–	–	–	–	–	–	–
Toilet structure	−0.06 (0.13)	−0.10 (0.13)	–	–	–	–	–	–	–	–	–
Toilet cleanliness	−0.12 (0.12)	0.06 (0.11)	–	–	–	–	–	–	–	–	–
Toilet location	−0.20 (0.17)	0.01 (0.17)	–	–	–	–	–	–	–	–	–
Household hygiene											
Presence of trash	0.03 (0.20)	−0.15 (0.19)	–	–	–	–	–	–	–	–	–
Presence of flies	0.19 (0.21)	0.15 (0.20)	–	–	–	–	–	–	–	–	–
Handwashing facility											
Location	–	–	−0.16 (0.14)	–	–	−0.24 (0.16)	–	–	0.05 (0.29)	–	–
Soap and water	–	–	0.16 (0.12)	–	–	0.13 (0.14)	–	–	0.20 (0.25)	–	–
Collection water storage											
Visible dirt	–	–	–	–	–	–	–	–	–	0.87 (0.62)	0.04 (0.68)
Container openings	–	–	–	–	–	–	–	–	–	0.65 (0.83)	−1.06 (0.69)
Handwashing water storage											
Visible dirt	–	–	–	–	–	–	–	–	–	−0.64 (0.65)	–
Container openings	–	–	–	–	–	–	–	–	–	0.10 (0.80)	–
Drinking water storage											
Visible dirt	–	–	–	–	–	–	–	–	–	–	0.00 (0.76)
Container openings	–	–	–	–	–	–	–	–	–	–	0.83 (0.65)
*R*^2^	0.10	0.04	0.38	0.05	0.02	0.47	0.01	0.05	0.29	0.16	0.18
*F* Statistic	1.52	0.59	11.43**	1.25	0.61	11.05**	0.21	0.86	2.03	0.70	0.77

* *P* < 0.05; ** *P* < 0.01. Significant correlations are highlighted in bold. Supplemental Table 1 in the Supplemental Material contains all the *P* values for the regression model.

Similarly, among households without running tap water, household characteristics and contamination in other compartments weakly or slightly explain *E. coli* contamination in environmental compartments (Table 3). The investigated factors in the models weakly explain *E. coli* levels in handwashing water and drinking water (*R*^2^ = 0.16 and 0.18, respectively). Household-level factors and contamination in other compartments show a stronger association with *E. coli* on hands after washing (*R*^2^ = 0.29, *P* = 0.08), but similar to households with running tap water, the strong association is due primarily to correlations with *E. coli* contamination on hands before handwashing (β = 0.33, SE = 0.13, *P* = 0.01).

The regression models for *E. coli* levels in soil and on hands before washing for all households are weakly explained by household characteristics and contamination in other compartments (Table 3). Specifically, *E. coli* on hands before washing is very weakly associated (*R*^2^ = 0.04) with modeled factors. Here, the contamination on hands before washing is associated with *E. coli* in soil with a small effect size (β = 0.17, SE = 0.09, *P* = 0.04). Similarly, *E. coli* on hands after washing is moderately associated (*R*^2^ = 0.38) with statistical significance for *E. coli* contamination on hands before washing (β = 0.13, SE = 0.06, *P* < 0.001) and in handwashing water (β = 0.50, SE = 0.06, *P* = 0.05).

## DISCUSSION

In households with young children in peri-urban Harare, Zimbabwe, we observed extensive *E. coli* contamination of environmental compartments, including drinking and handwashing water, soil, and hands before and after handwashing. The source and risk factors for *E. coli* contamination are not readily apparent from the data we collected on either household-level WASH risk factors or *E. coli* contamination of other compartments. One notable exception, however, is the presence of running tap water which is associated with significantly less *E. coli* contamination of drinking water, handwashing water, and hands after handwashing (but not soil nor hands before handwashing).

Although we observed extensive *E. coli* contamination in the environmental compartments, these concentrations are generally lower than have previously been reported in other studies. *Escherichia coli* contamination of stored drinking water is common in LMICs. Although the World Health Organization (WHO) standard for drinking water quality is < 1 CFU *E. coli*/100 mL, *E. coli* concentrations are frequently reported within the range of 0.9–3.3 log_10_ CFU/100 mL *E. coli*.^11,12,41,42,51–55^ In our study, 70.4% of samples (*N* = 100) exceed the WHO standard. Similarly, studies are increasingly highlighting *E. coli* contamination in the soil of 2.1–5.5 log_10_/g of soil.^10,13,27,33,38,56^ Furthermore, *E. coli* on hands is frequently reported to range from 0.4 to 4.5 log_10_ per two hands.^11,12,18,46,57^ The lower *E. coli* concentrations in soil and hand compartments observed in peri-urban Harare, Zimbabwe, could be associated with the presence of running tap water, low animal ownership rates, and presence of toilets, all factors that differed from the previous studies.^11,16,21,24,25,58–63^

Here, as elsewhere, availability of running tap water on premises is associated with reduced environmental contamination.^59,61,62,64^ Improved water sources significantly reduced the detection of *E. coli* virulence genes in stored drinking water in Tanzanian households.^11^ Furthermore, contamination of stored water with fecal indicator bacteria in Tanzanian communities was strongly associated with hand contamination.^13^ A systematic review of the impact of on-plot water sources has suggested a strong correlation with hygiene-associated diseases.^62^ On-plot water sources reduce the risk of contamination at the source, during collection, or during storage.

Soil contamination could not be explained by household-level WASH factors nor by concentrations of *E. coli* in other compartments. Nevertheless, two factors show a low but significant impact on *E. coli* levels in soil. Households with animals show significantly higher concentrations of *E. coli*. Animal fecal management has long been suspected as a contributor to fecal contamination in LMICs.^16,25,54,64^ In addition, lower *E. coli* contamination is observed in households with history of diarrheal disease. The inverse correlation between soil contamination and diarrhea incidence is both surprising and unexplainable. Diarrhea episodes could be potentially associated with improved fecal management practices and/or reduced mobility. While self-reporting on diarrhea is biased, there may be hidden covariates linking self-reporting diarrhea to other factors influencing soil contamination (such as socioeconomic status).^65^ Overall, it is perhaps unsurprising that *E. coli* in soil was not strongly linked to the factors reported here. Soil properties such as moisture content, sunlight, temperature, pH, the availability of resources (e.g., carbon), the physical characteristics of the soil (e.g., proportion of soil and silt), and the soil microbiota strongly influence *E. coli* concentrations; these soil properties may be the driving factors influencing soil *E. coli* concentrations.^13,66^ Future studies should consider identifying the most influential soil properties; here and elsewhere, the importance of moisture content, sunlight, and pH are clear, but uncertainty remains for other soil properties.^13,66^ Moreover, fecal indicator bacteria may be indigenous and/or naturalized in soil which could explain why *E. coli* in soil was not correlated with the reported factors.^27,32,33^

In terms of significance of the correlation between compartments and household-level factors, some factors that appear significant in the correlation matrix are not significant after Holm–Bonferroni family-wise error corrections. Specifically, in households without running tap water, *E. coli* levels on hands after handwashing are moderately associated with *E. coli* contamination of hands before washing (Spearman’s ρ = 0.30, *P* = 0.05) and the prevalence of diarrhea incidence (Spearman’s ρ = 0.26, *P* = 0.09). Furthermore, in all households, *E. coli* contamination on hands before handwashing is weakly associated with *E. coli* levels in soil (Spearman’s ρ = 0.15, *P* = 0.07). However, none of these associations are significant after applying the family-wise corrections (i.e., *adj. P* > 0.05). Future studies may consider further investigating these associations.

The study has some notable limitations. The limited sample size (97 for households with running tap water and 43 for households without) may account for the relative lack of statistical significance observed. We did not intend to subset the analyses by the presence of running tap water. Environmental sampling is costly and was the limiting factor in determining sample size. It is now clear from our data that the high variability in environmental *E. coli* contamination requires a larger sample size to increase the statistical power. Future studies incorporating environmental microbiological data should focus primarily on increasing sample sizes and/or covering larger/more diverse geographical regions. The inter-household variation of *E. coli* contamination within a single geographical cluster appears greater than variation due to environmental contamination and/or household-level risk factors within that cluster. A second limitation is our reliance on self-reporting for diarrheal incidence, which may introduce bias. For example, Manesh et al.^65^ describe improved recall among households with higher socioeconomic status; a covariate that may explain why we observed higher diarrheal disease incidence (expected from better recall) with lower *E. coli* soil contamination (expected from higher socioeconomic status). Furthermore, our study focused on *E. coli* as opposed to other enteric pathogens and/or fecal indicators. We focused on *E. coli* because of the relevance of *E. coli* pathovars in global diarrheal disease burden.^2^ However, other enteric pathogens could present different risk factors and/or associations with household-level factors and in environmental contamination levels. For example, environmental detection of norovirus and *Shigella* spp. (human pathogens) might be more closely linked to sanitation and household hygiene than rotavirus and diarrheagenic *E. coli* (which infect both humans and livestock) which may be more closely linked to animal ownership. Finally, regression models relied on coarse indices for assets and sanitation that weighted, equally, input data. For example, the asset indices gave equal weight to ownership of a radio as for a van. Weighting assets and/or sanitation characteristics may provide more accurate indices. The conceptual framework for the study suggests that *E. coli*, and fecal contamination generally, within various environmental compartments is influenced by contamination in other compartments and household-level factors (i.e., WASH). Our study demonstrates that this conceptual model explains only a small proportion of the total inter-household variation in compartmental *E. coli* contamination. The lack of an observed association may be because the conceptual model is wrong. Sources, fate, and transport of fecal contamination within a household may be minimally influenced by household-level factors. Fecal contamination may be more influenced by community-level factors (i.e., sanitation coverage, population density, community animal ownership, and climate). Related, inter-household variation in our field site may have been too low to observe impacts of household-level risk factors on *E. coli* contamination. Studies conducted over larger geographical regions that include more diverse communities may observe more significant associations. An alternative reason may be that *E. coli* is insufficient to elucidate the conceptual model of fecal contamination sources, fate, and transport. The microbial ecology of *E. coli*, including the ability to grow and persist in water and soil, may introduce noise that obscures household-level contributions to fecal contamination.^32,33^

Overall, our study demonstrates the need for public health interventions to reduce fecal contamination in the environment. It is, however, unclear where to invest for efficient interventions. We observed the reduction of *E. coli* contamination in drinking water and handwashing water and on hands after handwashing in the presence of ruining tap water. In addition, we report associations between the *E. coli* levels on hands after washing with handwashing water contamination and diarrhea incidence. Furthermore, we observed that animal ownership increases *E. coli* contamination in soil and the *E. coli* levels in soil are correlated with the contamination on hands before washing. Moreover, the detection of pathotype genes of EAEC in human feces and drinking water (data not shown) highlights the potential environmental transmissibility of pathogenic *E. coli* strains in Harare, Zimbabwe. These results suggest the interconnectedness of fecal indicator contamination between environmental compartments and the potential impact of household characteristics on drinking water quality and hand hygiene. Presently, there are several ongoing research trials investigating the impacts of WASH interventions on environmental fecal contamination.^4^ Our work highlights the complexity of *E. coli* contamination in low-income settings in LMICs. More, larger, studies are needed to find sources and exposure pathways of fecal contamination and to identify effective interventions.

## Supplementary Material

Supplemental Data and Table.
