# Supplementary material for: Escherichia coli Contamination across Multiple Environmental Compartments (Soil, Hands, Drinking Water, and Handwashing Water) in Urban Harare: Correlations and Risk Factors
Source: Am J Trop Med Hyg. 2018 Jan 22;98(3):803–13. doi: 10.4269/ajtmh.17-0521 (PMC5930891; doi:10.4269/ajtmh.17-0521)
Supplement: Supplementary file 1 [file tpmd170521.SD1.pdf]

SUPPLEMENTAL DATA: METHODS

Fecal samples. *Collection.* To collect human feces, fecal swabs with cotton tips (Faust, Schaffhausen, Switzerland) were given to the caregivers and they were instructed to provide a rectal swab sample collected in the privacy of their home or toilet. To collect chicken feces, feces that were fresh looking (wet and glossy) were collected aseptically.

Escherichia coli *isolation.* Swabs were stored on ice for up to 6 hours before processing in the laboratory at the University of Zimbabwe. To elute *E. coli*, 3 mL phosphate buffered saline solution was added to the swab tube and the tube was shaken by hand for 1 minute. The swab was streaked on TBX media to isolate presumptive *E. coli*. One isolate per sample was collected for human feces. Because of the low sample size of households with chickens (N = 17), between one and three isolates per sample was collected for chicken feces. Plates were then processed in the same manner as described for soil. At Eawag, a randomly selected subset of presumptive *E. coli* isolates (N = 54 for human feces, N = 35 for chicken feces) were identified with API-20E kits (bioMérieux) following the manufacturer's instructions.

SUPPLEMENTAL TABLE 1

P values for environmental compartments and hand hygiene models

	All households					With running tap water			Without running tap water		
	Soil	Hands before washing	Hands after washing	Handwashing water	Drinking water	Hands after washing	Handwashing water	Drinking water	Hands after washing	Handwashing water	Drinking water
Observations	140	140	139	140	140	96	97	97	43	42	42
Environmental compartments											
Soil	–	**0.047**	–	0.101	0.736	–	0.977	0.273	–	0.500	0.150
Handwashing water	–	–	**0.046**	–	–	0.768	–	–	0.054	–	–
Hands before washing	–	–	**< 0.001**	0.384	0.992	**< 0.001**	0.913	0.398	**0.015**	0.634	0.841
Diarrhea incidence											
Past 7 days	0.076	0.681	0.535	0.545	0.274	0.399	0.374	0.120	0.599	0.724	0.603
Asset ranking											
High	0.411	0.815	0.484	0.395	0.493	0.793	0.775	0.812	0.431	0.780	0.929
Low	0.764	0.711	0.406	0.946	0.496	0.0811	0.950	0.912	0.752	0.901	0.173
Sanitation											
Presence of animals	0.068	0.818	–	–	–	–	–	–	–	–	–
Toilet structure	0.700	0.444	–	–	–	–	–	–	–	–	–
Toilet cleanliness	0.327	0.583	–	–	–	–	–	–	–	–	–
Toilet location	0.243	0.975	–	–	–	–	–	–	–	–	–
Household hygiene											
Presence of trash	0.898	0.414	–	–	–	–	–	–	–	–	–
Presence of flies	0.341	0.444	–	–	–	–	–	–	–	–	–
Handwashing facility											
Location	–	–	0.249	–	–	0.134	–	–	0.871	–	–
Soap and water	–	–	0.182	–	–	0.374	–	–	0.419	–	–
Collection water storage											
Visible dirt	–	–	–	–	–	–	–	–	–	0.169	0.955
Container openings	–	–	–	–	–	–	–	–	–	0.432	0.134
Handwashing water storage											
Visible dirt	–	–	–	–	–	–	–	–	–	0.330	–
Container openings	–	–	–	–	–	–	–	–	–	0.909	–
Drinking water storage											
Visible dirt	–	–	–	–	–	–	–	–	–	–	0.998
Container openings	–	–	–	–	–	–	–	–	–	–	0.215
Model *P* value	0.148	0.818	**< 0.001**	0.290	0.691	**< 0.001**	0.960	0.511	0.079	0.705	0.646

Significant correlations are highlighted in bold.
